# Chromosome-Scale Assembly of the Complete Genome Sequence of Porcisia hertigi, Isolate C119, Strain LV43

**DOI:** 10.1128/MRA.00651-21

**Published:** 2021-10-14

**Authors:** Hatim Almutairi, Michael D. Urbaniak, Michelle D. Bates, Waleed S. Al-Salem, Rod J. Dillon, Paul A. Bates, Derek Gatherer

**Affiliations:** a Division of Biomedical & Life Sciences, Faculty of Health & Medicine, Lancaster University, Lancaster, United Kingdom; b Ministry of Health, Riyadh, Saudi Arabia; University of California, Riverside

## Abstract

Porcisia hertigi is a parasitic kinetoplastid first isolated from porcupines (Coendou rothschildi) in central Panama in 1965. We present the complete genome sequence of *P. hertigi*, isolate C119, strain LV43, sequenced using combined short- and long-read technologies. This complete genome sequence will contribute to our knowledge of the parasitic genus *Porcisia*.

## ANNOUNCEMENT

Porcisia hertigi was discovered in Central Panama (1) in porcuines (Coendou rothschildi) and later in Costa Rica (2). Initially classified as Leishmania hertigi, subsequent phylogenetic studies resulted in its reclassification into a new genus, *Porcisia* (3), within the subfamily *Leishmaniinae* (4). To date, only a partial assembly of Porcisia deanei (5) (strain TCC258) is available within this genus. We now assemble the complete genome sequence of *Porcisia hertigi*, strain LV43, isolate C119 (WHO code MCOE/PA/1965/C119;LV43), isolated from a porcupine in Panama in 1965. This new complete genome sequence will contribute to our understanding of the evolution of both the genus *Porcisia* and the subfamily *Leishmaniinae*.

Parasites were grown using an *in vitro* culture system previously developed for Leishmania (Mundinia) orientalis axenic amastigotes (6), in Schneider’s insect medium at 26°C as promastigotes, then in M199 medium supplemented with 10% FCS, 2% stable human urine, 1% basal medium Eagle vitamins, and 25 μg/ml gentamicin sulfate, with subpassage to fresh medium every 4 days to sustain the parasite growth and viability. DNA was extracted and purified using a Qiagen DNeasy blood and tissue kit with the spin column protocol, according to the manufacturer’s instructions. The extracted DNA concentration was assessed using a Qubit fluorometer, microplate reader, and agarose gel electrophoresis. All sequencing libraries were based on the same extracted DNA sample to avoid any inconsistency.

Short-read library construction and sequencing were contracted to (i) BGI (Shenzhen, China) for DNBSEQ libraries, producing paired-end reads (270 bp and 500 bp) using the Illumina HiSeq platform, and (ii) Aberystwyth University (Aberystwyth, UK) for TruSeq Nano DNA libraries, producing paired-end reads (300 bp) using the Illumina MiSeq platform. We performed long-read library preparation and sequencing according to the Nanopore protocol (SQK-LSK109) on R9 flow cells (FLO-MIN106). The read quality was assessed using MultiQC (7), incorporating the use of FastQC for the Illumina short reads and pycoQC for the Nanopore long reads.

We assembled the long reads using Flye (8), with default parameters, to generate chromosome-scale scaffolds. Then, using Minimap2 (9) and SAMtools (10), we mapped the short reads onto the assembled scaffolds to compensate for erroneous bases within the long reads and create consensus sequences. After polishing the assembly using Pilon (11), another round of consensus short-read mapping was performed. Then, we removed duplicated contigs and sorted the remainder according to length using Funannotate (12). Finally, we separated the chimeric sequences and performed scaffolding using RaGOO (13) with the Leishmania major strain Friedlin genome (GenBank accession number GCA_000002725.2) (14) as a reference guide, aligning all 36 chromosomes for our assembly, with the exception of 38 unplaced contigs totaling 1,892,991 bp.

**FIG 1:**
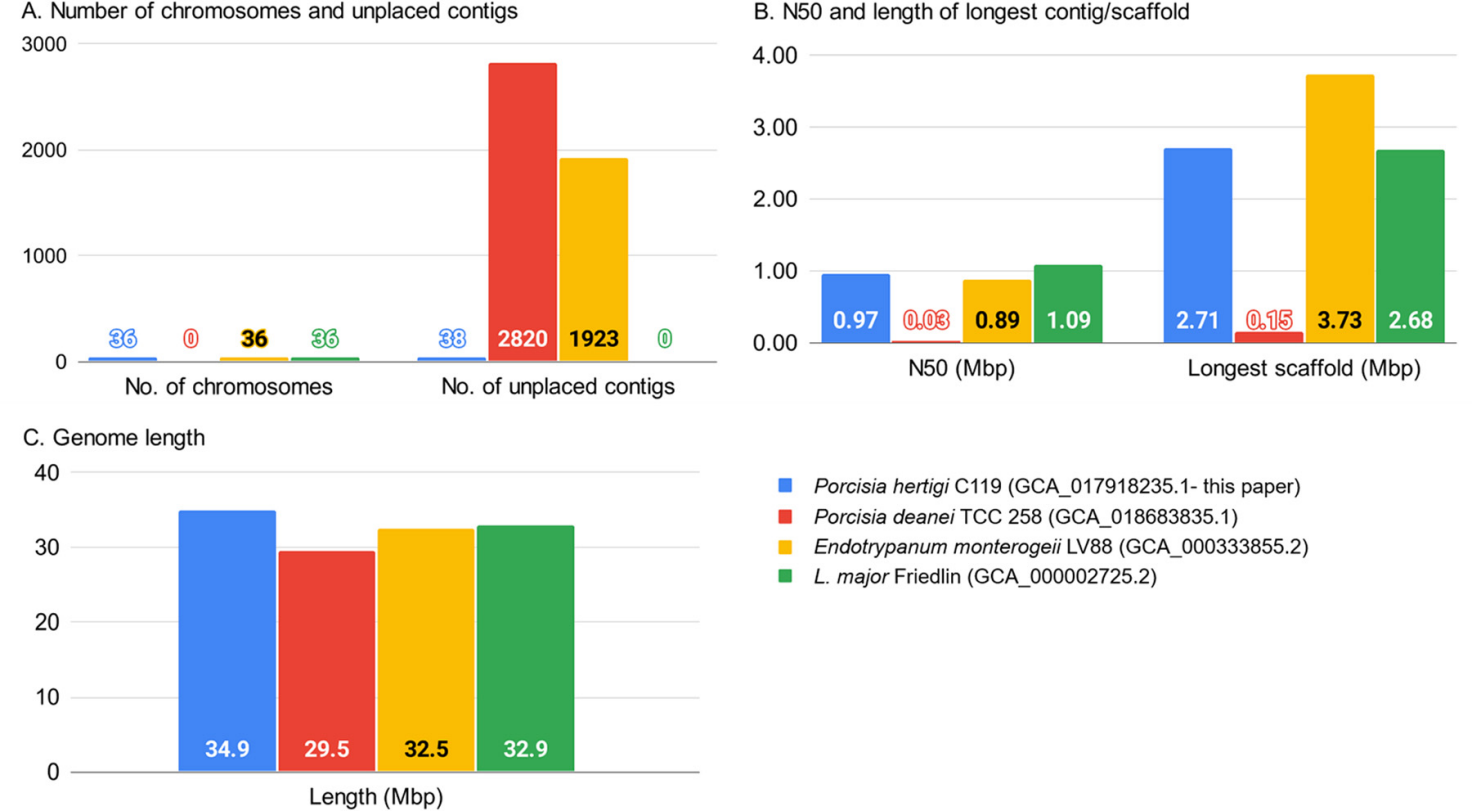
Assembly comparison of *P. hertigi* LV43 with Endotrypanum monterogeii LV88, L. major Friedlin, and *P. deanei* TCC258.

The analysis workflow for assembly and annotation was performed using Snakemake (15) and is available online for reproducibility purposes (https://github.com/hatimalmutairi/LGAAP), including the software versions and parameters used (16). Figure 1 compares our assembly with other complete genomes.

We assessed the assembly completeness using BUSCO (17), with the lineage data set for the phylum *Euglenozoa*, containing 130 single-copy orthologs from 31 species, finding that 126 of these were present (96.92% completeness). We carried out functional annotation and prediction using the MAKER2 (18) annotation pipeline in combination with AUGUSTUS (19) gene prediction software. Table 1 shows additional summary metrics for the sequencing, assembly, and annotation.

**TABLE 1:** Detailed summary metrics of the genome sequencing, assembly, and annotation for *P. hertigi* LV43

Feature(s)	Metric(s)
Total no. of reads	27,383,632
No. of MiSeq reads	3,785,008
No. of HiSeq reads	23,382,754
No. of MinION reads (read *N*_50_ [bp])	215,870 (20,520)
Genome size (Gbp)	13.41
Genome coverage (×)	177.1
Total no. of scaffolds	74
Genome size (bp)	34,958,538
*N*_50_ (bp)	967,170
GC content (%)	56.00
No. of *N*s (% of genome)	320 (0.001)
No. of genes	7,891
Gene density (genes/Mb)	225.7
No. of exons	8,270
Mean gene length (bp)	1,908
Total length of CDSs^*a*^ (Mb [% of genome])	14.70 (42.06)

a CDSs, coding DNA sequences.

### Data availability.

The assembly and annotations are available under GenBank assembly accession number GCA_017918235.1. The master record for the whole-genome sequencing project is available at JAFJZO000000000.1. The raw sequence reads are available under BioProject accession number PRJNA691541.
